# Potent Enhancement of HIV-1 Replication by Nef in the Absence of SERINC3 and SERINC5

**DOI:** 10.1128/mBio.01071-19

**Published:** 2019-06-11

**Authors:** Yuanfei Wu, Balaji Olety, Eric R. Weiss, Elena Popova, Hikaru Yamanaka, Heinrich Göttlinger

**Affiliations:** a Department of Molecular, Cell and Cancer Biology, University of Massachusetts Medical School, Worcester, Massachusetts, USA; King’s College London; Columbia University/HHMI

**Keywords:** Nef, SERINC5, human immunodeficiency virus, infectivity, virus replication

## Abstract

It has recently emerged that HIV-1 Nef counteracts the antiviral host proteins SERINC3 and SERINC5. In particular, SERINC5 inhibits the infectivity of progeny virions when incorporated. SERINC3 and SERINC5 are also counteracted by the unrelated murine leukemia virus glycosylated Gag (glycoGag) protein, which possesses a potent Nef-like activity on HIV-1 infectivity. We now report that a minimal glycoGag termed glycoMA can fully substitute for Nef in promoting HIV-1 replication in Jurkat T lymphoid cells, indicating that Nef enhances replication in these cells mainly by counteracting SERINCs. In contrast, the SERINC antagonist glycoMA was unable to substitute for Nef in MOLT-3 T lymphoid cells, in which HIV-1 replication was highly dependent on Nef, and remained so even in the absence of SERINC3 and SERINC5. As in MOLT-3 cells, glycoMA was unable to substitute for Nef in stimulating HIV-1 replication in primary human cells. Although the ability of Nef mutants to promote HIV-1 replication in MOLT-3 cells correlated with the ability to engage endocytic machinery and to downregulate CD4, Nef nevertheless rescued virus replication under conditions where CD4 downregulation did not occur. Taken together, our observations raise the possibility that Nef triggers the endocytosis of a novel antiviral factor that is active against both laboratory-adapted and primary HIV-1 strains.

## Abstract (precis)

The Nef protein of HIV-1 and the unrelated glycoGag protein of a murine leukemia virus similarly prevent the uptake of antiviral host proteins called SERINC3 and SERINC5 into HIV-1 particles, which enhances their infectiousness. We now show that although both SERINC antagonists can in principle similarly enhance HIV-1 replication, glycoGag is unable to substitute for Nef in primary human cells and in a T cell line called MOLT-3. In MOLT-3 cells, Nef remained crucial for HIV-1 replication even in the absence of SERINC3 and SERINC5. The pronounced effect of Nef on HIV-1 spreading in MOLT-3 cells correlated with the ability of Nef to engage cellular endocytic machinery and to downregulate the HIV-1 receptor CD4 but nevertheless persisted in the absence of CD4 downregulation. Collectively, our results provide evidence for a potent novel restriction activity that affects even relatively SERINC-resistant HIV-1 isolates and is counteracted by Nef.

## INTRODUCTION

Nef is an accessory protein of primate lentiviruses that is crucial for efficient virus replication and progression to AIDS in a monkey model (1) and also determines the pathogenicity of human immunodeficiency virus type 1 (HIV-1) (2). HIV-1 Nef enhances viral growth in cell lines and in primary human CD4^+^ T cells, but the effects can be quite modest (3). However, relatively robust effects of Nef on HIV-1 spreading have been observed in primary CD4^+^ T cells that were infected before mitogenic stimulation (4, 5). Indeed, the ability to stimulate HIV-1 replication in primary human cells under these conditions is widely conserved among HIV and simian immunodeficiency virus (SIV) Nef proteins (6). Other conserved activities of Nef include the engagement of cellular endocytic machinery for the downregulation of CD4 and of major histocompatibility class I molecules (7, 8). Of these activities, the downregulation of CD4 by Nef was found to play a crucial role in HIV-1 replication in primary T cells (9).

Most HIV and SIV Nef proteins also enhance the specific infectivity of progeny virions (6, 10–12). The infectivity enhancement function of Nef requires its expression in virus producer cells (12, 13) and is compromised if these are depleted of clathrin or dynamin 2 (14). Although Nef counteracts effects of high levels of CD4 on HIV-1 release and infectivity (15, 16), Nef can enhance HIV-1 infectivity even in the absence of CD4 (12) or in the presence of a CD4 that is resistant to downregulation by Nef (13). The magnitude of the effect of Nef on infectivity is determined by variable regions of the HIV-1 envelope (Env) glycoprotein, and some primary HIV-1 Envs are relatively poorly responsive to Nef (17).

HIV-1 infectivity is also potently enhanced by the accessory glycosylated Gag (glycoGag) protein of the gammaretrovirus Moloney murine leukemia virus (MLV), which does not downregulate CD4 (18). MLV glycoGag is a type II transmembrane protein with an amino-terminal cytosolic domain that is solely responsible for its Nef-like activity (19). Although glycoGag is entirely unrelated to Nef, its effect on HIV-1 infectivity, like that of Nef, depends on clathrin-mediated endocytosis and is determined by the V2 and V3 regions of Env (17, 19).

Nef and glycoGag both downregulate the multipass transmembrane proteins SERINC3 and SERINC5 from the cell surface and prevent their incorporation into HIV-1 virions (20–22). Furthermore, the unrelated S2 accessory protein of equine infectious anemia virus (EIAV), which can complement the infectivity defect of Nef-deficient (Nef^−^) HIV-1 virions, also prevents the incorporation of SERINC3 and SERINC5 into virus particles (23). In particular, virion-associated SERINC5 can dramatically reduce the infectivity of HIV-1 virions (20–22), and the ability of Nef to counteract SERINC5 is widely conserved among primate lentiviruses (24). Although certain primary HIV-1 Envs are relatively SERINC5 resistant, virion-associated SERINC5 nevertheless increases the sensitivity of such Envs to some neutralizing antibodies (25). Specifically, SERINC5 appears to enhance the sensitivity of HIV-1 to neutralizing antibodies that target the gp41 membrane-proximal extracellular region (MPER) (25, 26). Apart from restricting HIV-1 infectivity, SERINC5 significantly inhibits the specific infectivities of certain gammaretroviral particles in an Env-dependent manner, and these effects are also counteracted by MLV glycoGag or EIAV S2 (27).

SERINC knockout (KO) and reconstitution experiments have demonstrated that SERINC3 and SERINC5 together restrict both HIV-1 infectivity and HIV-1 replication in Jurkat TAg (JTAg cells). We now show that a minimal glycoGag (termed glycoMA) supports HIV-1 replication as efficiently as Nef in JTAg cells, consistent with the notion that SERINCs fully account for the attenuation of Nef^−^ HIV-1 in these cells.

However, the SERINC antagonist glycoMA was unable to rescue HIV-1 replication in MOLT-3 cells, a T lymphoid cell line with relatively low SERINC5 levels in which HIV-1 spreading is nevertheless highly dependent on Nef. In these cells, Nef potently enhanced the replication of HIV-1 isolates encoding both SERINC-sensitive and SERINC-resistant Envs. Furthermore, the requirement for Nef persisted in double-knockout MOLT-3 cells lacking SERINC3 and SERINC5 (MOLT-3 *S3/5* KO cells). Although the ability of Nef to promote virus replication in MOLT-3 cells correlated with its ability to downregulate CD4, Nef rescued HIV-1 replication even under conditions where CD4 downregulation did not occur. Nef-deficient progeny virions produced in MOLT-3 cells were remarkably poorly infectious, possibly explaining why Nef was crucial for virus spreading in these cells. Importantly, as in MOLT-3 cells, HIV-1 replication in primary human peripheral blood mononuclear cells (PBMC) that were infected prior to stimulation depended on Nef and could not be rescued by glycoMA. Thus, MOLT-3 cells may provide a relevant experimental system to understand how Nef enhances HIV-1 replication.

## RESULTS

### MLV glycoMA can substitute for Nef in HIV-1 replication.

We previously reported that Nef is critical for the spread of HIV-1_NL4-3_ in JTAg cells but dispensable in double-knockout JTAg cells lacking *SERINC3* and *SERINC5* (20). Importantly, Nef once again became critical after reconstitution of SERINC3 and SERINC5 expression in the double-KO cells (20). Furthermore, more permissive CD4^high^ versions of the parental, double-knockout, and reconstituted double-knockout JTAg cells yielded similar results (20). Because MLV glycoGag and a fully active N-terminal portion termed glycoMA share the ability of Nef to counteract SERINC3 and SERINC5 and to enhance HIV-1 progeny virion infectivity (17–21), we asked whether glycoMA can also promote HIV-1 replication in the presence of SERINC3 and SERINC5. To this end, we infected CD4^high^ JTAg cells with equal amounts of wild-type (WT) (Nef-positive [Nef^+^]) or Nef^−^ HIV-1_NL4-3_ or with NL4-3/glycoMA, a glycoMA^+^ version of HIV-1_NL4-3_ that contains a sequence encoding glycoMA in place of *nef* (19).

**FIG 1:**
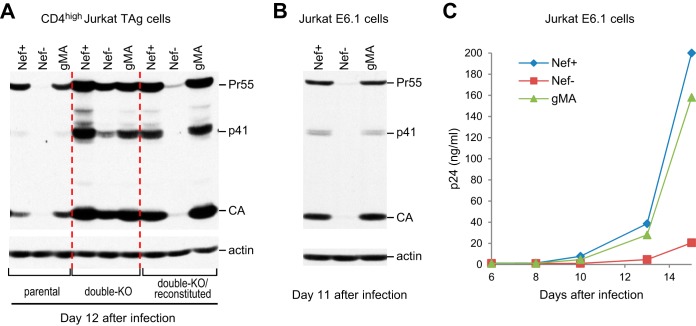
MLV glycoMA can substitute for Nef in promoting HIV-1 replication in Jurkat cells. (A) Western blots showing the effects of Nef and glycoMA on HIV-1 spreading in parental CD4^high^ JTAg cells, double knockout cells lacking SERINC3 and SERINC5, and SERINC3- and SERINC5-reconstituted double-knockout cells. The cells were infected with equal amounts (2 ng/ml p24) of Nef^+^, Nef^−^, or glycoMA^+^ HIV-1_NL4-3_, and cell lysates were examined with anti-CA and anti-actin 12 days after infection. A duplicate experiment gave similar results. (B and C) Nef and glycoMA similarly enhance HIV-1_NL4-3_ replication in Jurkat E6.1 cells, as examined by Western blotting of cell lysates 11 days after infection (B) and by monitoring p24 accumulation in the supernatants (C). The cells were infected with 0.2 ng p24/ml. The data in panels B and C are from independent experiments.

As previously reported (20), Nef enhanced the replication of HIV-1_NL4-3_ in CD4^high^ JTAg cells, as determined by examining the levels of Gag protein expression in the infected cultures by Western blotting (Fig. 1A). Notably, Gag expression levels on day 12 after infection with Nef^+^ or glycoMA^+^ HIV-1_NL4-3_ were comparable (Fig. 1A), implying that glycoMA was as capable of enhancing HIV-1 replication as Nef itself. As expected, Nef^−^ HIV-1_NL4-3_ replicated far more efficiently in double-knockout CD4^high^ JTAg cells lacking SERINC3 and SERINC5, but Nef again became critical for replication when SERINC3 and SERINC5 expression in the double-knockout cells was restored (Fig. 1A). Importantly, glycoMA rescued virus replication in the reconstituted double-knockout cells to a similar extent as Nef (Fig. 1A), confirming that glycoMA was fully capable of counteracting the restriction to HIV-1 spreading imposed by SERINC3 and SERINC5.

We also examined whether glycoMA affects HIV-1 replication in Jurkat E6.1 cells, which are considerably more permissive for HIV-1_NL4-3_ than JTAg or even CD4^high^ JTAg cells (20). Nevertheless, we have observed that the spread of HIV-1_NL4-3_ in Jurkat E6.1 cells is significantly accelerated by Nef when the cells are infected with relatively small amounts of input virus (20). Although we used a slightly higher concentration of input virus (200 pg/ml p24) in the two independent experiments shown in Fig. 1B and C, we again observed a marked enhancement of HIV-1 spreading in Jurkat E6.1 cells by Nef, as determined by measuring Gag expression in the infected cells (Fig. 1B) or p24 antigen release over time (Fig. 1C). HIV-1 spreading was enhanced to a comparable extent by glycoMA (Fig. 1B and C), indicating that as in JTAg cells, the effect of Nef on HIV-1_NL4-3_ replication in Jurkat E6.1 cells is largely due to its ability to counteract SERINCs.

### The ability of glycoGag to substitute for Nef is cell type dependent.

**FIG 2:**
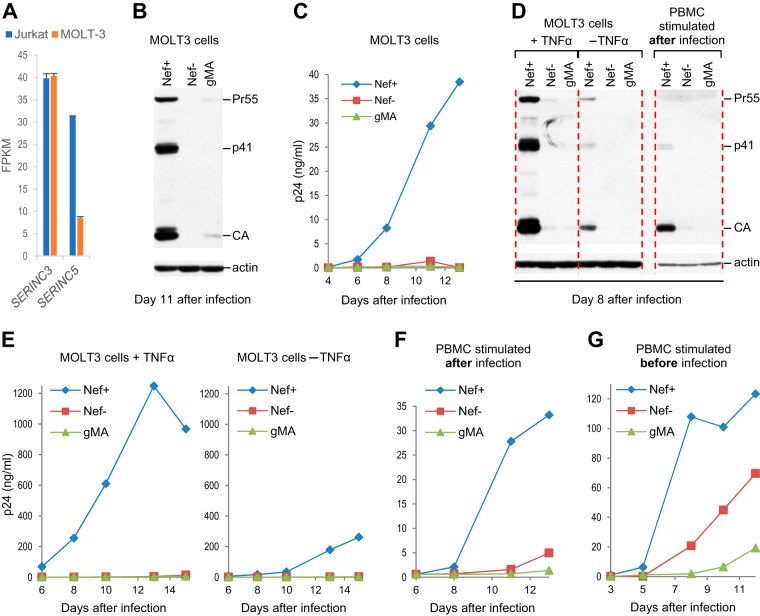
Potent enhancement of HIV-1 replication in MOLT-3 cells and PBMC by Nef but not glycoMA. (A) *SERINC* mRNA expression in Jurkat E6.1 and MOLT-3 cells quantified by transcriptome sequencing (RNA-seq) as fragments per kilobase of transcript per million mapped reads (FPKM) (*n* = 2). (B) Nef but not glycoMA (gMA) potently enhances HIV-1_NL4-3_ replication in MOLT-3 cells. Gag expression at day 11 after infection with equal amounts (0.2 ng/ml p24) of Nef^+^, Nef^−^, or glycoMA^+^ HIV-1_NL4-3_ was examined by Western blotting. (C) Virus replication in MOLT-3 cells infected with the same viruses (at 2 ng/ml p24) monitored by a p24 ELISA. (D) Virus replication in MOLT-3 cells and in PBMC examined in parallel by Western blotting of cell lysates with anti-CA. MOLT-3 cells stimulated or not with 100 ng/ml TNF-α were infected with Nef^+^, Nef^−^, or glycoMA^+^ HIV-1_NL4-3_ (0.2 ng/ml p24 each). Unstimulated PBMC were infected with the same viruses (at 0.25 ng/ml p24) and stimulated with PHA on day 4 after infection. (E and F) Virus replication in the same cultures monitored by a p24 ELISA. (G) Virus replication in prestimulated PBMC monitored by a p24 ELISA after infection with Nef^+^, Nef^−^, or glycoMA^+^ HIV-1_NL4-3_ (0.25 ng/ml p24 each). This experiment was performed twice. The data in panels B to D are from independent experiments. The results in panels E and F were confirmed in independent experiments.

We also examined the role of Nef in HIV-1 replication in MOLT-3 T lymphoid cells, which express about 4-fold-lower levels of *SERINC5* mRNA than Jurkat cells (Fig. 2A). Nevertheless, we observed that HIV-1_NL4-3_ replication in MOLT-3 cells is highly dependent on Nef (Fig. 2B and C). Furthermore, unlike in Jurkat E6.1 cells, which were infected in parallel (Fig. 1B), WT (Nef^+^) HIV-1_NL4-3_ replicated far more efficiently in MOLT-3 cells than did the glycoMA^+^ version, as judged from the amount of Gag expressed in the cells on day 11 after infection (Fig. 2B). In a separate experiment, in which virus replication was monitored by measuring the release of p24 antigen over time, the replication of HIV-1_NL4-3_ in MOLT-3 cells was similarly profoundly impaired when *nef* was either disrupted or replaced by a sequence encoding glycoMA (Fig. 2C).

Because Nef has been reported to prime T cells for activation (28–30), we also examined the effects of Nef and glycoMA on HIV-1 replication in tumor necrosis factor alpha (TNF-α)-stimulated MOLT-3 cells. Notably, it has been shown that stimulation with TNF-α at 10 ng/ml is sufficient to potently induce HIV-1 expression in latently infected Jurkat cells (31). Although the replication of WT (Nef^+^) HIV-1_NL4-3_ in MOLT-3 cells stimulated with 100 ng/ml TNF-α was greatly accelerated compared to its replication in unstimulated cells, Nef^−^ and glycoMA^+^ HIV-1_NL4-3_ did not replicate, even in TNF-α-stimulated MOLT-3 cells (Fig. 2D and E). These observations imply that Nef can profoundly accelerate HIV-1 replication even in activated cells.

Nef enhances the spread of HIV-1 in primary human target cells, particularly when these cells are infected prior to mitogenic stimulation (4–6). To determine whether glycoMA mimics the effect of Nef on HIV-1 replication in primary cells, unstimulated human PBMC were infected immediately after isolation and stimulated with phytohemagglutinin (PHA) 4 days later. Interestingly, under these conditions, the replication of the Nef^+^, Nef^−^, and glycoMA^+^ viruses in primary cells resembled that in MOLT-3 cells, which were infected in parallel with a similarly small amount of input virus (Fig. 2D to F). Specifically, while HIV-1_NL4-3_ (Nef^+^) clearly replicated in primary cells following PHA stimulation, the replication of both Nef^−^ HIV-1_NL4-3_ and NL4-3/glycoMA was highly attenuated (Fig. 2D and F). Consistent with a previous study (9), Nef also enhanced the replication of T cell-tropic HIV-1_NL4-3_ in prestimulated PBMC, but glycoMA did not (Fig. 2G). We conclude that glycoMA mimicked the effect of Nef on HIV-1 replication in Jurkat but not in MOLT-3 cells or in PBMC.

### Replication of SERINC5-resistant HIV-1 in MOLT-3 cells also depends on Nef.

The observation that glycoMA expressed in *cis* rescued the replication of Nef-deficient HIV-1 in Jurkat cells is consistent with the notion that Nef accelerates HIV-1 replication in these cells by counteracting SERINCs. Conversely, the inability of glycoMA to substitute for Nef in MOLT-3 cells suggested that the profound restriction of HIV-1 replication that we observed in these cells in the absence of Nef was not merely caused by SERINCs. To examine this issue, we generated variants of HIV-1_NL4-3_, termed NL-JRFL and NL-ADA, which have the *env* gene replaced with that from the JRFL and ADA primary HIV-1 isolates, respectively. In contrast to Env_NL4-3_, which is highly responsive to Nef and glycoMA, Env_JRFL_ and Env_ADA_ are poorly Nef responsive (17), indicating that they are relatively resistant to SERINCs. In support of this notion, the depletion of SERINC3 and SERINC5 in virus-producing JTAg cells has little effect on the infectivity of progeny virions bearing Env_JRFL_, even though it profoundly increases the infectivity of virions bearing Nef-responsive Envs (20). Furthermore, there is direct evidence that even overexpressed SERINC5 has relatively little effect on the infectivity of HIV-1 pseudovirions bearing Env_JRFL_ (21).

**FIG 3:**
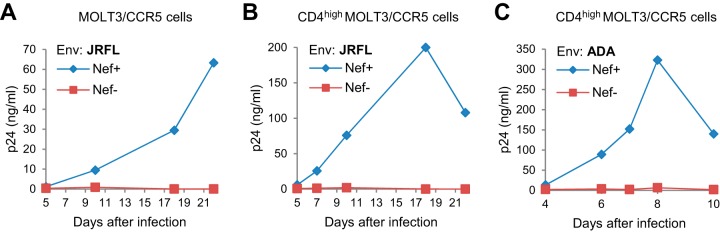
Replication of SERINC5-resistant HIV-1 in MOLT-3 cells depends on Nef. (A and B) Virus replication in MOLT-3/CCR5 cells (A) or CD4^high^ MOLT-3/CCR5 cells (B) infected with equal amounts (1 ng p24/ml) of NL-JRFL or NL-JRFL/nef^−^. HIV-1 replication was monitored by a p24 ELISA. (C) Virus replication in CD4^high^ MOLT-3/CCR5 cells infected with equal amounts (10 ng p24/ml) of NL-ADA or NL-ADA/nef^−^. The results in panels B and C were confirmed in experiments performed with different amounts of input virus.

Because Env_JRFL_ and Env_ADA_ are R5 tropic, we generated MOLT-3 cells expressing CCR5 by retroviral transduction. Following infection with Nef^+^ and Nef^−^ versions of NL-JRFL, we observed that the Nef^+^ version replicated in these cells, whereas the Nef^−^ version did not (Fig. 3A). Since even the Nef^+^ version replicated relatively slowly, we also used retroviral transduction to generate CD4^high^ MOLT-3 cells expressing CCR5. As expected, these cells were more permissive for Nef^+^ NL-JRFL, but Nef^−^ NL-JRFL still failed to replicate over an observation period of more than 3 weeks (Fig. 3B). Additionally, we observed that only a Nef^+^ version of NL-ADA replicated in these cells (Fig. 3C), even though a relatively large amount of input virus (10 ng/ml p24) was used in this experiment. These observations indicate that in MOLT-3 cells, even the replication of HIV-1 strains that are relatively resistant to SERINCs is highly dependent on Nef.

### Nef is required for efficient HIV-1 replication in MOLT-3 cells lacking SERINC3 and SERINC5.

**FIG 4:**
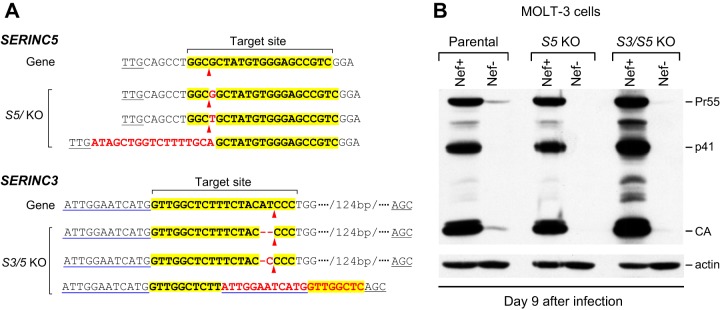
Nef is required for efficient HIV-1 replication in MOLT-3 cells lacking SERINC3 and SERINC5. (A) Mutant SERINC alleles identified in SERINC5 knockout and SERINC3/5 double-knockout clones. The sgRNA target sites are highlighted, and the predicted Cas9 target sites are indicated by arrowheads. Inserted nucleotides are in red. One of the mutated *SERINC3* alleles in MOLT-3 *S3/5* KO cells has a large deletion that removes the splice site at the 3′ end of the targeted exon. All other mutations cause frameshifts. No WT alleles were detected in any of the KO clones. (B) Western blots showing the effects of Nef on HIV-1 spreading in MOLT-3 cells lacking SERINC5 (*S5* KO) or SERINC3 and SERINC5 (*S3/5* KO). The cells were infected with equal amounts (0.2 ng/ml p24) of Nef^+^ or Nef^−^ HIV-1_NL4-3_. This experiment was performed twice.

The observation that glycoMA did not mimic the effect of Nef on HIV-1_NL4-3_ replication in MOLT-3 cells raised the possibility that the profound restriction of Nef-deficient HIV-1 in these cells was not primarily due to SERINC3 and SERINC5, which are counteracted by both Nef and glycoMA. To examine this possibility, we knocked out *SERINC5* in MOLT-3 cells by CRISPR/Cas9-mediated gene editing and used the resulting cells to additionally knock out *SERINC3* (Fig. 4A). The ability of HIV-1_NL4-3_ to replicate remained highly dependent on Nef both in knockout MOLT-3 cells lacking SERINC5 and in double-knockout MOLT-3 cells lacking SERINC3 and SERINC5 (Fig. 4B). These results support the notion that MOLT-3 cells express another restriction factor that is counteracted by Nef.

### Replication of Nef mutants in MOLT-3 cells.

**FIG 5:**
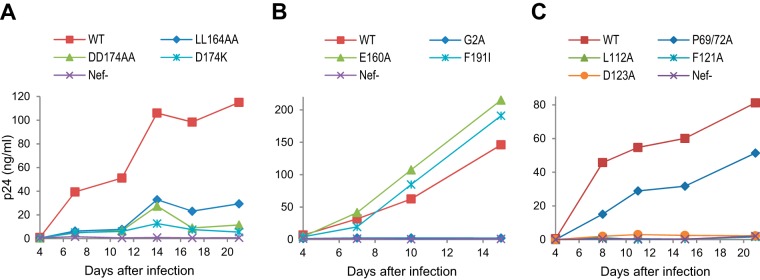
Replication of Nef mutants in MOLT-3 cells. (A) Effects of mutations that abrogate AP-2 binding and/or CD4 downregulation by Nef. (B) Effects of mutations that disrupt AP-1 binding (E160A), PAK2 binding (F191I), or Nef myristylation (G2A). (C) Effects of mutations that disrupt interactions with Src kinases (P69/72A) or dynamin 2 (L112A, F121A, and D123A). MOLT-3 cells were infected with 5 ng (A) or 2 ng (B and C) p24/ml, and virus replication was monitored by a p24 ELISA.

A previous analysis of a panel of Nef mutants revealed a strong genetic correlation between the ability of Nef to enhance HIV-1 replication in activated primary CD4^+^ T cells and its ability to downregulate CD4 (9). For instance, the L164/165A and D174/175A mutations, which have been shown to abrogate the ability of Nef to downregulate CD4 without affecting Nef expression levels (9, 32–34), significantly delayed replication in primary cells although not as severely as a null mutation in *nef* (9). Similarly, the L164/165A and D174/175A mutations significantly impaired the replication of HIV-1_NL4-3_ in MOLT-3 cells, while a null mutation in *nef* completely prevented the replication of HIV-1_NL4-3_ in this experiment (Fig. 5A). We also tested the effect of a lysine substitution at position 174 of Nef (D174K), which has been reported to completely disrupt CD4 downregulation without compromising Nef expression or its ability to affect T cell receptor signaling (35, 36). As shown in Fig. 5A, the D174K mutant replicated as poorly in MOLT-3 cells as the D174/175A mutant.

Together with Nef residue E160, the L164-L165 dileucine motif within the central loop of Nef fits the consensus for a [DE]XXXL[LI]-type sorting signal recognized by clathrin adaptor protein (AP) complexes (32, 37). Indeed, an E160A mutation in Nef abrogated binding to AP-1 and AP-3 hemicomplexes (37). Nevertheless, the E160A mutation had little if any effect on the ability of Nef to downregulate CD4 (32, 33, 37), possibly because this activity of Nef depends on AP-2 (37). As shown in Fig. 5B, the E160A mutation also had no effect on the ability of Nef to enhance the replication of HIV-1_NL4-3_ in MOLT-3 cells.

The replication of HIV-1_NL4-3_ in MOLT-3 cells was not affected by the F191I mutation in Nef (Fig. 5B), which specifically eliminates the association of Nef with p21-activated kinase 2 (PAK2) without compromising other activities, such as CD4 downregulation (38). In contrast, HIV-1_NL4-3_ replication in MOLT-3 cells was severely impaired by the G2A mutation (Fig. 5B). The G2A mutation has been reported to disrupt Nef myristylation without affecting Nef expression (39), to impair the downregulation of CD4 by Nef (40), and to compromise HIV-1 replication in primary T cells (9).

PXXP motifs in HIV-1 Nef have been implicated in interactions with the SH3 domains of Src kinases and in the enhanced replication of Nef^+^ viruses in PBMC (41). However, in another study, a P69/P72A mutation in Nef had no effect on HIV-1 replication in primary CD4^+^ cells and only partially impaired CD4 downregulation by Nef (9). As shown in Fig. 5C, the P69/P72A mutation moderately delayed the replication of HIV-1_NL4-3_ in MOLT-3 cells. More drastic replication defects were observed with single amino acid substitutions of surface-exposed Nef core domain residues (L112A, F121A, and D123A) that have been shown to impair the interaction of Nef with the endocytic fission factor dynamin 2 without affecting Nef expression levels (14) (Fig. 5C). Of note, Nef residues L112, F121, and D123 are all individually critical for CD4 downregulation (42, 43). Taken together, these observations in the MOLT-3 cell system were consistent with a role of CD4 downregulation in the enhancement of HIV-1 replication by Nef, as was proposed previously (9).

### Nef can profoundly enhance HIV-1 replication in the absence of CD4 downregulation.

**FIG 6:**
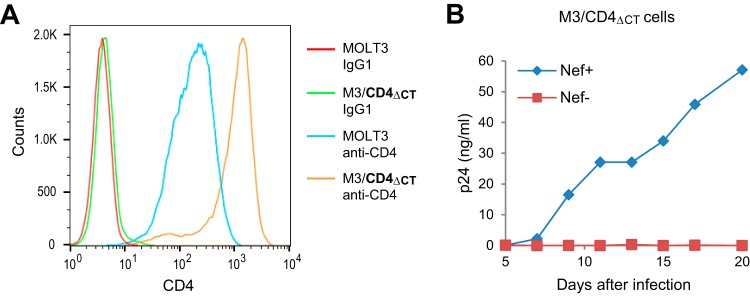
Nef enhances HIV-1 replication in MOLT-3 cells expressing high levels of a Nef-resistant CD4. (A) CD4 surface levels on parental MOLT-3 cells and on MOLT-3 cells stably transduced with a retroviral vector expressing a Nef-resistant CD4 (M3/CD4_ΔCT_ cells). (B) Virus replication in M3/CD4_ΔCT_ cells monitored by a p24 ELISA after infection with equal amounts (2 ng p24/ml) of Nef^+^ or Nef^−^ HIV-1_NL4-3_.

Because the enhancement of HIV-1 replication in MOLT-3 cells by Nef was impaired by mutations that are known to impair its ability to downregulate CD4, we examined whether Nef is capable of enhancing virus replication in MOLT-3 cells expressing a Nef-resistant CD4 molecule. To avoid the downregulation of cell surface CD4 by Nef, we used retroviral transduction to generate MOLT-3 cells that express high levels of a truncated CD4 molecule (M3/CD4_ΔCT_ cells). The CD4_ΔCT_ mutant lacks the 25 C-terminal residues of the cytoplasmic domain (44), which is required for Nef-induced CD4 downregulation (45). In particular, the CD4_ΔCT_ molecule lacks a membrane-proximal dileucine motif in the cytoplasmic domain that is critical for Nef-induced endocytosis (40). As shown in Fig. 6A, the CD4 levels on M3/CD4_ΔCT_ cells exceed the endogenous levels on MOLT-3 cells by nearly 10-fold, indicating that most of the CD4 on M3/CD4_ΔCT_ cells lacked a cytoplasmic domain required for Nef-induced downregulation. Nevertheless, WT (Nef^+^) HIV-1_NL4-3_ clearly replicated in these cells, whereas a Nef^−^ version showed no sign of replication during a 20-day observation period (Fig. 6B).

**FIG 7:**
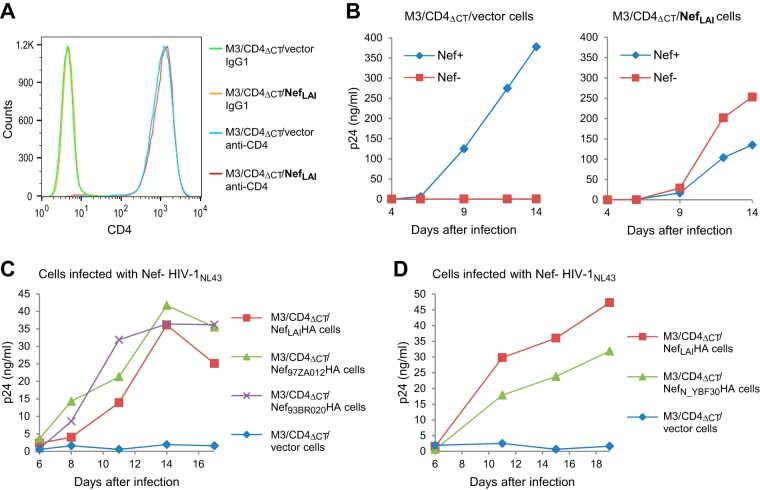
Nef enhances HIV-1 replication in MOLT-3 cells in the absence of CD4 downregulation. (A) CD4 surface levels on M3/CD4_ΔCT_ cells stably transduced with the empty pCX4pur retroviral vector (M3/CD4_ΔCT_/vector cells) or a version expressing Nef_LAI_ (M3/CD4_ΔCT_/Nef_LAI_ cells). (B) Virus replication in M3/CD4_ΔCT_/vector and M3/CD4_ΔCT_/Nef_LAI_ cells monitored by a p24 ELISA after infection with equal amounts (2 ng p24/ml) of Nef^+^ or Nef^−^ HIV-1_NL4-3_. The effect of Nef in *trans* on Nef^−^ HIV-1_NL4-3_ was confirmed in an independent experiment. (C and D) Replication of Nef^−^ HIV-1_NL4-3_ in M3/CD4_ΔCT_ cells stably expressing various group M Nef proteins (C) or a group N Nef protein (D).

To formally exclude the possibility that significant downregulation of CD4 by Nef occurred in infected M3/CD4_ΔCT_ cells because of the presence of endogenous CD4, we expressed Nef in M3/CD4_ΔCT_ cells from a retroviral vector. As shown in Fig. 7A, cells stably transduced with a vector encoding Nef_LAI_ (M3/CD4_ΔCT_/Nef_LAI_ cells) expressed exactly the same amount of surface CD4 as cells transduced with the empty vector (M3/CD4_ΔCT_/vector cells). As expected, Nef^+^ but not Nef^−^ HIV-1_NL4-3_ replicated in M3/CD4_ΔCT_/vector cells (Fig. 7B, left). In marked contrast, Nef^−^ HIV-1_NL4-3_ replicated in M3/CD4_ΔCT_/Nef_LAI_ cells even better than Nef^+^ HIV-1_NL4-3_ (Fig. 7B, right). Thus, Nef was able to rescue the replication of Nef^−^ HIV-1 when provided in *trans*, even though it had no effect on CD4 surface levels.

Since Nef_LAI_ is from a laboratory-adapted strain, we also generated M3/CD4_ΔCT_ cells stably transduced with retroviral vectors encoding the Nef proteins of primary group M and group N HIV-1 isolates, none of which affected CD4 or CXCR4 surface levels (data not shown). Nef^−^ HIV-1_NL4-3_ replicated with comparable efficiencies in M3/CD4_ΔCT_ cells expressing the group M Nef proteins Nef_LAI_ (subtype B), Nef_97ZA012_ (subtype C), and Nef_93BR020_ (subtype F) in *trans* (Fig. 7C). Furthermore, the replication of Nef^−^ HIV-1_NL4-3_ was only slightly delayed in M3/CD4_ΔCT_ cells expressing the Nef protein of group N isolate YBF30 rather than a group M Nef protein (Fig. 7D). Importantly, replication was again negligible when no Nef was provided in *trans* (Fig. 7C and D). We conclude that the ability of Nef to enhance HIV-1 replication in the absence of CD4 downregulation is conserved among widely divergent HIV-1 Nef proteins.

### Nef but not glycoMA enhances HIV-1 infectivity in MOLT-3 cells.

**FIG 8:**
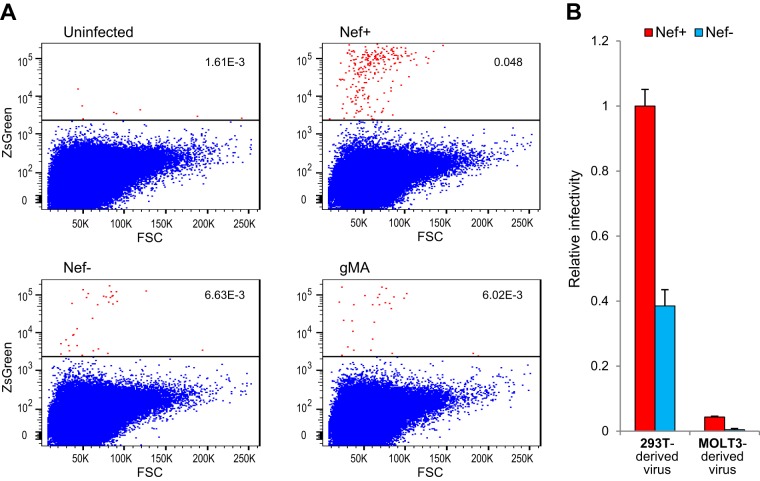
Nef but not glycoMA enhances the infectivity of HIV-1 produced in MOLT-3 cells. (A) Dot blots showing ZsGreen expression in MOLT-3/ZsGreen reporter cells after infection with equal amounts of Nef^+^, Nef^−^, or glycoMA^+^ HIV-1_NL4-3_ produced in MOLT-3 cells. FSC, forward scatter. (B) HIV-1 virions produced in MOLT-3 cells are relatively poorly infectious. Relative infectivities of Nef^+^ and Nef^−^ HIV-1_NL4-3_ produced in 293T or MOLT-3 cells were measured using TZM-bl indicator cells.

To determine the effects of Nef and glycoMA on the infectivity of virions produced in MOLT-3 cells, infections were started with relatively large amounts of input virus, and virus replication was allowed to proceed for about 3 weeks. The cell culture medium was then replaced with fresh medium, and virus was harvested 24 h later and used to infect MOLT-3/ZsGreen indicator cells after normalization for p24 antigen. MOLT-3/ZsGreen cells contain a reporter gene encoding ZsGreen-NLS that is *trans*-activated by Tat upon infection with HIV-1 (46). To limit virus replication in the MOLT-3 indicator cells to a single round, an entry inhibitor (AMD3100) was added after overnight incubation. After further incubation to allow ZsGreen expression, infected (ZsGreen-positive) cells were quantified by flow cytometry. As shown in Fig. 8A, the specific infectivity of MOLT-3-derived WT (Nef^+^) HIV-1_NL4-3_ measured on MOLT-3 indicator cells was about 10-fold higher than that of Nef^−^ or glycoMA^+^ HIV-1_NL4-3_ produced in the same cells. Thus, glycoMA does not share the ability of Nef to enhance HIV-1 infectivity in MOLT-3 cells.

However, even the infectivity of the WT virus appeared to be very low. To examine whether this was due to the use of MOLT-3-based indicator cells, we instead used HeLa-derived TZM-bl indicator cells in another experiment. Furthermore, we compared the infectivities of Nef^+^ and Nef^−^ HIV-1_NL4-3_ produced by infected MOLT-3 cells to equal amounts of virus produced by transfected 293T cells. Remarkably, compared to 293T cell-derived virus, the specific infectivity of WT (Nef^+^) HIV-1_NL4-3_ was more than 20-fold lower when produced in MOLT-3 cells, and the specific infectivity of Nef^−^ HIV-1_NL4-3_ was more than 80-fold lower (Fig. 8B). These observations indicate that Nef^−^ virus produced in MOLT-3 cells is barely infectious, which may explain why Nef is crucial for virus propagation in these cells.

## DISCUSSION

In principle, Nef can enhance HIV-1 replication simply by counteracting SERINC3 and SERINC5, as implied by SERINC knockout and reconstitution experiments in JTAg cells (20). In the present study, we have obtained evidence supporting this notion by showing that a minimal version of the SERINC antagonist glycoGag can fully substitute for Nef in enhancing HIV-1 replication in JTAg and Jurkat E6.1 cells, which express large amounts of endogenous SERINC5 (21). Although MLV glycoGag and the glycoMA derivative are entirely unrelated to Nef, they share its ability to counteract the effects of SERINC3 and SERINC5 on HIV-1 infectivity (20).

Indeed, glycoGag and glycoMA are more potent than Nef in enhancing the infectivities of HIV-1 virions bearing Envs that are highly sensitive to SERINCs, such as Env_NL4-3_ (17, 20). We were therefore surprised that glycoMA was essentially unable to substitute for Nef in promoting the spread of HIV-1_NL4-3_ in MOLT-3 cells, a T cell line in which we find HIV-1 replication to be particularly dependent on Nef, even though *SERINC5* mRNA levels are about 4-fold lower than those in Jurkat cells. Indeed, Nef-deficient HIV-1_NL4-3_ was highly attenuated even in MOLT-3 cells lacking SERINC3 and SERINC5, whereas Nef^+^ HIV-1_NL4-3_ replicated vigorously. Remarkably, a dramatic requirement for Nef for virus replication was also observed in MOLT-3 cells stimulated with TNF-α. Although TNF-α significantly accelerated the replication kinetics of WT (Nef^+^) HIV-1_NL4-3_ in MOLT-3 cells, and led to very high levels of virus production, a Nef^−^ version or a version encoding glycoMA instead of Nef still failed to replicate.

The effects of Nef and of SERINCs on HIV-1 infectivity are determined by HIV-1 Env, and at least some primary Envs appear to be far less affected by SERINCs than laboratory-adapted Envs (17, 20, 21). For instance, HIV-1 infectivity conferred by the Env protein of the R5-tropic primary strain JRFL was only marginally enhanced by Nef or the simultaneous depletion of SERINC3 and SERINC5 (17, 20) and was only moderately sensitive to overexpressed SERINC5 (21). Therefore, effects of Nef on HIV-1 replication that depend on its ability to counteract SERINCs would be expected to be much less significant in the presence of relatively SERINC-resistant Envs, such as Env_JRFL_ or Env_ADA_, than in the presence of the highly SERINC-sensitive Env_NL4-3_. However, in MOLT-3 cells, the replication of variants of HIV-1_NL4-3_ that encode Env_JRFL_ or Env_ADA_ was as strongly dependent on Nef as that of authentic HIV-1_NL4-3_. These findings show that MOLT-3 cells tightly restrict both SERINC-sensitive and SERINC-resistant viruses that lack Nef, which raises the possibility that these cells express a Nef-sensitive antiviral host factor that targets both laboratory-adapted and primary HIV-1 strains.

The L164/165A and D174/175A mutations in Nef, which abrogate AP-2 binding by Nef (47), significantly impaired HIV-1 replication in MOLT-3 cells, consistent with the notion that Nef is needed for the downregulation of an antiviral host factor in these cells. While the L164/165 dileucine motif is required for the binding of Nef to several clathrin adaptor complexes, the D174/175 diacidic motif is required only for the interaction with AP-2 (47). Our analysis of a panel of Nef mutants also suggested that the ability of Nef to rescue HIV-1 replication in MOLT-3 cells correlates with its ability to downregulate CD4. Interestingly, a strong genetic correlation between CD4 downregulation by Nef and the efficiency of HIV-1 replication in primary T cells has been noted previously (9). It has also been shown that high levels of CD4 on infected cells sequester HIV-1 Env and dramatically reduce progeny virion infectivity, an effect that is counteracted by Nef (16). We have therefore explored the possibility that CD4 downregulation by Nef contributes to its enhancement of virus replication in MOLT-3 cells. However, MOLT-3 cells that predominantly expressed a truncated CD4 that is resistant to Nef-mediated downregulation remained permissive for Nef^+^ HIV-1. Furthermore, a panel of widely divergent HIV-1 group M and group N Nef proteins, when stably expressed in *trans* in these cells, rescued the replication of Nef^−^ HIV-1 but did not affect cell surface CD4 levels. Taken together, these observations indicate that the ability to rescue HIV-1 replication in MOLT-3 cells is highly conserved among HIV-1 Nef proteins and does not depend on CD4 downregulation.

Our results indicate that Nef^−^ virus produced in MOLT-3 cells is very poorly infectious, which may account for the pronounced replication defect of Nef^−^ HIV-1 in these cells. Consistent with this notion, Nef enhanced both the infectivity of MOLT-3-derived virus and virus replication, whereas glycoMA did neither. Also, the inability of glycoMA to enhance the infectivity of Nef^−^ virus produced in MOLT-3 cells suggests that SERINCs are not primarily responsible for its very low specific infectivity.

As in MOLT-3 cells, Nef was critical for HIV-1 replication in primary human PBMC that were infected prior to stimulation, and the potent SERINC antagonist glycoMA was unable to substitute for Nef. Of note, this observation is consistent with the recent finding that SIVcol Nef does not efficiently enhance HIV-1 replication in human primary cells, even though it induces the efficient degradation of SERINC5 (48). Taken together, our observations indicate that the ability of Nef to antagonize SERINCs fully accounts for its effect on HIV-1_NL4-3_ replication in some cell lines but not in MOLT-3 cells or in primary human CD4^+^ T cells. Furthermore, the comparably potent effects of Nef but not glycoMA in MOLT-3 cells and in PBMC suggest that MOLT-3 cells provide a relevant model system to examine the role of Nef in HIV-1 replication.

## MATERIALS AND METHODS

### HIV-1 proviral constructs.

NL4-3/Nefstop is a *nef*-deficient variant of the pNL4-3 infectious proviral clone (20). NL4-3/glycoMA was also described previously (19). The infectious proviral clones NL-JRFL, NL-JRFL/nef^−^, NL-ADA, and NL-ADA/nef^−^ were generated by replacing a KpnI-BamHI fragment of pNL4-3 or NL4-3/nef^−^ (14) with the corresponding fragment from the pSVIIIenv-based Env_JRFL_ (49) and Env_ADA_ (50) expression vectors. NL4-3/nef^−^ harbors a frameshift in *nef* (14). Mutations in the *nef* gene of pNL4-3 were introduced by inserting mutant *nef* sequences derived from subviral constructs between unique BamHI and XhoI or XhoI and NcoI sites.

### Retroviral vectors.

The codon-optimized human *CCR5* gene synCCR5 (51) was cloned into the retroviral vectors pCXbsr (52) and pCX4pur (53). The coding sequence for human CD4 was cloned into pCXbsr. The pCXbsrCD4_ΔCT_ retroviral vector, which encodes a human CD4 that lacks most of the cytoplasmic domain, was described previously (46). HIV-1 group M *nef* genes were amplified from previously described expression plasmids (14) and cloned into pCX4pur. A codon-optimized gene encoding the Nef protein of group N isolate YBF30 (GenBank accession number CAA06817) was synthesized and cloned into pCX4pur.

### Cells.

Jurkat E6.1 and MOLT-3 cells were obtained from the ATCC. TZM-bl indicator cells were obtained from the AIDS Research and Reference Reagent Program, NIAID, NIH. CD4^high^ JTAg cells, double-knockout CD4^high^ JTAg cells lacking SERINC3 and SERINC5, and double-knockout CD4^high^ JTAg cells reconstituted with SERINC3 and SERINC5 expression cassettes were described previously (20).

MOLT-3 cells lacking SERINC5 were obtained as described previously (20), by transiently transfecting an expression plasmid for a single-guide RNA (sgRNA) targeting an exon within the *SERINC5* gene into MOLT-3 cells by nucleofection, along with a plasmid expressing Cas9. To obtain double-knockout cells, MOLT-3 *S5* KO cells were cotransfected with an sgRNA targeting the *SERINC3* gene and the Cas9 expression plasmid, as described previously (20). The sites targeted by the sgRNAs are depicted in Fig. 4A. Clones obtained by limiting dilution were prescreened by PCR amplification of the targeted regions of the genome and digestion of the PCR products with HaeII (for *SERINC5*) or BtsCI (for *SERINC3*), as described previously (20). In cases where no WT allele could be detected by restriction enzyme analysis, the PCR products were cloned into pCR-Blunt II-TOPO (Life Technologies), and approximately 20 independent clones were sequenced.

MOLT-3/ZsGreen indicator cells were described previously (46). MOLT-3/CCR5 cells were generated by retroviral transduction with pCXbsr-synCCR5 and selection with blasticidin. CD4^high^ MOLT-3/CCR5 cells were obtained by transduction with pCXbsrCD4 and selection with blasticidin, followed by transduction with pCX4pur-synCCR5 and selection with puromycin. M3/CD4_ΔCT_ cells were made by transducing MOLT-3 cells with pCXbsrCD4_ΔCT_, followed by selection with blasticidin. M3/CD4_ΔCT_ cells were subsequently transduced with the empty vector pCX4pur or with versions encoding various Nef proteins, followed by selection with puromycin.

### Virus replication studies.

Virus was produced by transiently transfecting 293T cells with replication-competent HIV-1 proviral clones. Virus-containing supernatants were passed through 0.45-μm filters, normalized for p24 antigen with an HIV-1 p24 enzyme-linked immunosorbent assay (ELISA) kit (PerkinElmer or XpressBio), and used to infect target cells. T lymphoid cells (2 × 10^5^) were infected in T25 flasks in 5 ml medium. PBMC were isolated from the blood of healthy donors by Ficoll-Hypaque density gradient centrifugation, seeded into 6-well plates at a density of 10 × 10^6^ cells/well in 4 ml medium, and immediately infected at a p24 concentration of 0.25 ng/ml. On day 4 after infection, the cells were stimulated with 1 μg/ml PHA (Sigma). The next day, the PHA-containing culture medium was replaced with medium containing 10 U/ml interleukin 2 (Roche Applied Science). Alternatively, PBMC were prestimulated with 1 μg/ml PHA immediately after isolation and infected 24 h later in the presence of 10 U/ml interleukin 2. Virus replication was examined by comparing Gag protein expression levels in infected cells by Western blotting using anti-CA antibody 183-H12-5C and by measuring p24 antigen in the culture supernatants by a p24 ELISA.

### Analysis of virus infectivity.

MOLT-3 cells chronically infected with NL4-3, NL4-3/Nefstop, or NL4-3/glycoMA were washed and resuspended in fresh medium. Supernatants harvested 24 h later were clarified by low-speed centrifugation, filtered through 0.45-μm filters, normalized for p24 antigen, and used to infect MOLT-3/ZsGreen clone 45 cells, which turn bright green upon HIV-1 infection (46). To limit virus replication to a single cycle, the entry inhibitor AMD3100 (5 μM) was added 16 h later. After another 2 days, the cells were stained with Live/Dead fixable far-red dead cell stain (Invitrogen) and fixed with 4% paraformaldehyde, and ZsGreen expression by live cells was analyzed on a Becton, Dickinson LSRII flow cytometer.

Alternatively, supernatants containing progeny virions produced by transiently transfected 293T cells or chronically infected MOLT-3 cells were used to infect TZM-bl indicator cells in duplicate. On day 3 postinfection, the indicator cells were lysed, β-galactosidase activity induced as a consequence of infection was measured, and values were normalized for the amount of p24 antigen present in the supernatants used for infection, as described previously (54).

## References

[1] Kestler HW III, Ringler DJ, Mori K, Panicali DL, Sehgal PK, Daniel MD, Desrosiers RC 1991 Importance of the nef gene for maintenance of high virus loads and for development of AIDS. Cell 65:651–662. doi:10.1016/0092-8674(91)90097-I. 2032289

[2] Deacon NJ, Tsykin A, Solomon A, Smith K, Ludford-Menting M, Hooker DJ, McPhee DA, Greenway AL, Ellett A, Chatfield C, Lawson VA, Crowe S, Maerz A, Sonza S, Learmont J, Sullivan JS, Cunningham A, Dwyer D, Dowton D, Mills J 1995 Genomic structure of an attenuated quasi species of HIV-1 from a blood transfusion donor and recipients. Science 270:988–991. doi:10.1126/science.270.5238.988. 7481804

[3] Kim S, Ikeuchi K, Byrn R, Groopman J, Baltimore D 1989 Lack of a negative influence on viral growth by the nef gene of human immunodeficiency virus type 1. Proc Natl Acad Sci U S A 86:9544–9548. doi:10.1073/pnas.86.23.9544. 2687883 PMC298533

[4] Miller MD, Warmerdam MT, Gaston I, Greene WC, Feinberg MB 1994 The human immunodeficiency virus-1 nef gene product: a positive factor for viral infection and replication in primary lymphocytes and macrophages. J Exp Med 179:101–113. doi:10.1084/jem.179.1.101. 8270859 PMC2191317

[5] Spina CA, Kwoh TJ, Chowers MY, Guatelli JC, Richman DD 1994 The importance of nef in the induction of human immunodeficiency virus type 1 replication from primary quiescent CD4 lymphocytes. J Exp Med 179:115–123. doi:10.1084/jem.179.1.115. 7903679 PMC2191324

[6] Munch J, Rajan D, Schindler M, Specht A, Rucker E, Novembre FJ, Nerrienet E, Muller-Trutwin MC, Peeters M, Hahn BH, Kirchhoff F 2007 Nef-mediated enhancement of virion infectivity and stimulation of viral replication are fundamental properties of primate lentiviruses. J Virol 81:13852–13864. doi:10.1128/JVI.00904-07. 17928336 PMC2168858

[7] Garcia JV, Miller AD 1991 Serine phosphorylation-independent downregulation of cell-surface CD4 by nef. Nature 350:508–511. doi:10.1038/350508a0. 2014052

[8] Schwartz O, Marechal V, Le Gall S, Lemonnier F, Heard JM 1996 Endocytosis of major histocompatibility complex class I molecules is induced by the HIV-1 Nef protein. Nat Med 2:338–342. doi:10.1038/nm0396-338. 8612235

[9] Lundquist CA, Tobiume M, Zhou J, Unutmaz D, Aiken C 2002 Nef-mediated downregulation of CD4 enhances human immunodeficiency virus type 1 replication in primary T lymphocytes. J Virol 76:4625–4633. doi:10.1128/JVI.76.9.4625-4633.2002. 11932428 PMC155097

[10] Chowers MY, Spina CA, Kwoh TJ, Fitch NJ, Richman DD, Guatelli JC 1994 Optimal infectivity in vitro of human immunodeficiency virus type 1 requires an intact nef gene. J Virol 68:2906–2914. 8151761 10.1128/jvi.68.5.2906-2914.1994 PMC236779

[11] Miller MD, Warmerdam MT, Page KA, Feinberg MB, Greene WC 1995 Expression of the human immunodeficiency virus type 1 (HIV-1) nef gene during HIV-1 production increases progeny particle infectivity independently of gp160 or viral entry. J Virol 69:579–584. 7983759 10.1128/jvi.69.1.579-584.1995 PMC188614

[12] Aiken C, Trono D 1995 Nef stimulates human immunodeficiency virus type 1 proviral DNA synthesis. J Virol 69:5048–5056. 7541845 10.1128/jvi.69.8.5048-5056.1995 PMC189322

[13] Schwartz O, Marechal V, Danos O, Heard JM 1995 Human immunodeficiency virus type 1 Nef increases the efficiency of reverse transcription in the infected cell. J Virol 69:4053–4059. 7539505 10.1128/jvi.69.7.4053-4059.1995 PMC189139

[14] Pizzato M, Helander A, Popova E, Calistri A, Zamborlini A, Palu G, Gottlinger HG 2007 Dynamin 2 is required for the enhancement of HIV-1 infectivity by Nef. Proc Natl Acad Sci U S A 104:6812–6817. doi:10.1073/pnas.0607622104. 17412836 PMC1871867

[15] Ross TM, Oran AE, Cullen BR 1999 Inhibition of HIV-1 progeny virion release by cell-surface CD4 is relieved by expression of the viral Nef protein. Curr Biol 9:613–621. doi:10.1016/S0960-9822(99)80283-8. 10375525

[16] Lama J, Mangasarian A, Trono D 1999 Cell-surface expression of CD4 reduces HIV-1 infectivity by blocking Env incorporation in a Nef- and Vpu-inhibitable manner. Curr Biol 9:622–631. doi:10.1016/S0960-9822(99)80284-X. 10375528

[17] Usami Y, Gottlinger H 2013 HIV-1 Nef responsiveness is determined by Env variable regions involved in trimer association and correlates with neutralization sensitivity. Cell Rep 5:802–812. doi:10.1016/j.celrep.2013.09.028. 24209751 PMC3864691

[18] Pizzato M 2010 MLV glycosylated-Gag is an infectivity factor that rescues Nef-deficient HIV-1. Proc Natl Acad Sci U S A 107:9364–9369. doi:10.1073/pnas.1001554107. 20439730 PMC2889076

[19] Usami Y, Popov S, Gottlinger HG 2014 The Nef-like effect of murine leukemia virus glycosylated gag on HIV-1 infectivity is mediated by its cytoplasmic domain and depends on the AP-2 adaptor complex. J Virol 88:3443–3454. doi:10.1128/JVI.01933-13. 24403584 PMC3957955

[20] Usami Y, Wu Y, Gottlinger HG 2015 SERINC3 and SERINC5 restrict HIV-1 infectivity and are counteracted by Nef. Nature 526:218–223. doi:10.1038/nature15400. 26416733 PMC4600458

[21] Rosa A, Chande A, Ziglio S, De Sanctis V, Bertorelli R, Goh SL, McCauley SM, Nowosielska A, Antonarakis SE, Luban J, Santoni FA, Pizzato M 2015 HIV-1 Nef promotes infection by excluding SERINC5 from virion incorporation. Nature 526:212–217. doi:10.1038/nature15399. 26416734 PMC4861059

[22] Matheson NJ, Sumner J, Wals K, Rapiteanu R, Weekes MP, Vigan R, Weinelt J, Schindler M, Antrobus R, Costa AS, Frezza C, Clish CB, Neil SJ, Lehner PJ 2015 Cell surface proteomic map of HIV infection reveals antagonism of amino acid metabolism by Vpu and Nef. Cell Host Microbe 18:409–423. doi:10.1016/j.chom.2015.09.003. 26439863 PMC4608997

[23] Chande A, Cuccurullo EC, Rosa A, Ziglio S, Carpenter S, Pizzato M 2016 S2 from equine infectious anemia virus is an infectivity factor which counteracts the retroviral inhibitors SERINC5 and SERINC3. Proc Natl Acad Sci U S A 113:13197–13202. doi:10.1073/pnas.1612044113. 27803322 PMC5135340

[24] Heigele A, Kmiec D, Regensburger K, Langer S, Peiffer L, Sturzel CM, Sauter D, Peeters M, Pizzato M, Learn GH, Hahn BH, Kirchhoff F 2016 The potency of Nef-mediated SERINC5 antagonism correlates with the prevalence of primate lentiviruses in the wild. Cell Host Microbe 20:381–391. doi:10.1016/j.chom.2016.08.004. 27631701 PMC5098270

[25] Beitari S, Ding S, Pan Q, Finzi A, Liang C 2017 Effect of HIV-1 Env on SERINC5 antagonism. J Virol 91:e02214-16. doi:10.1128/JVI.02214-16. 27928004 PMC5286904

[26] Sood C, Marin M, Chande A, Pizzato M, Melikyan GB 2017 SERINC5 protein inhibits HIV-1 fusion pore formation by promoting functional inactivation of envelope glycoproteins. J Biol Chem 292:6014–6026. doi:10.1074/jbc.M117.777714. 28179429 PMC5392591

[27] Ahi YS, Zhang S, Thappeta Y, Denman A, Feizpour A, Gummuluru S, Reinhard B, Muriaux D, Fivash MJ, Rein A 2016 Functional interplay between murine leukemia virus glycogag, Serinc5, and surface glycoprotein governs virus entry, with opposite effects on gammaretroviral and ebolavirus glycoproteins. mBio 7:e01985-16. doi:10.1128/mBio.01985-16. PMC5120145 27879338

[28] Wang JK, Kiyokawa E, Verdin E, Trono D 2000 The Nef protein of HIV-1 associates with rafts and primes T cells for activation. Proc Natl Acad Sci U S A 97:394–399. doi:10.1073/pnas.97.1.394. 10618429 PMC26674

[29] Fenard D, Yonemoto W, de Noronha C, Cavrois M, Williams SA, Greene WC 2005 Nef is physically recruited into the immunological synapse and potentiates T cell activation early after TCR engagement. J Immunol 175:6050–6057. doi:10.4049/jimmunol.175.9.6050. 16237100

[30] Markle TJ, Philip M, Brockman MA 2013 HIV-1 Nef and T-cell activation: a history of contradictions. Future Virol 8:391–404. doi:10.2217/fvl.13.20. PMC3810967 24187576

[31] Tyagi M, Pearson RJ, Karn J 2010 Establishment of HIV latency in primary CD4^+^ cells is due to epigenetic transcriptional silencing and P-TEFb restriction. J Virol 84:6425–6437. doi:10.1128/JVI.01519-09. 20410271 PMC2903277

[32] Craig HM, Pandori MW, Guatelli JC 1998 Interaction of HIV-1 Nef with the cellular dileucine-based sorting pathway is required for CD4 down-regulation and optimal viral infectivity. Proc Natl Acad Sci U S A 95:11229–11234. doi:10.1073/pnas.95.19.11229. 9736718 PMC21624

[33] Bresnahan PA, Yonemoto W, Ferrell S, Williams-Herman D, Geleziunas R, Greene WC 1998 A dileucine motif in HIV-1 Nef acts as an internalization signal for CD4 downregulation and binds the AP-1 clathrin adaptor. Curr Biol 8:1235–1238. doi:10.1016/S0960-9822(07)00517-9. 9811606

[34] Aiken C, Krause L, Chen YL, Trono D 1996 Mutational analysis of HIV-1 Nef: identification of two mutants that are temperature-sensitive for CD4 downregulation. Virology 217:293–300. doi:10.1006/viro.1996.0116. 8599214

[35] Iafrate AJ, Bronson S, Skowronski J 1997 Separable functions of Nef disrupt two aspects of T cell receptor machinery: CD4 expression and CD3 signaling. EMBO J 16:673–684. doi:10.1093/emboj/16.4.673. 9049297 PMC1169669

[36] Mariani R, Kirchhoff F, Greenough TC, Sullivan JL, Desrosiers RC, Skowronski J 1996 High frequency of defective nef alleles in a long-term survivor with nonprogressive human immunodeficiency virus type 1 infection. J Virol 70:7752–7764. 8892896 10.1128/jvi.70.11.7752-7764.1996 PMC190845

[37] Coleman SH, Madrid R, Van Damme N, Mitchell RS, Bouchet J, Servant C, Pillai S, Benichou S, Guatelli JC 2006 Modulation of cellular protein trafficking by human immunodeficiency virus type 1 Nef: role of the acidic residue in the ExxxLL motif. J Virol 80:1837–1849. doi:10.1128/JVI.80.4.1837-1849.2006. 16439540 PMC1367136

[38] Foster JL, Molina RP, Luo T, Arora VK, Huang Y, Ho DD, Garcia JV 2001 Genetic and functional diversity of human immunodeficiency virus type 1 subtype B Nef primary isolates. J Virol 75:1672–1680. doi:10.1128/JVI.75.4.1672-1680.2001. 11160665 PMC114076

[39] Guy B, Riviere Y, Dott K, Regnault A, Kieny MP 1990 Mutational analysis of the HIV nef protein. Virology 176:413–425. doi:10.1016/0042-6822(90)90011-F. 2111956

[40] Aiken C, Konner J, Landau NR, Lenburg ME, Trono D 1994 Nef induces CD4 endocytosis: requirement for a critical dileucine motif in the membrane-proximal CD4 cytoplasmic domain. Cell 76:853–864. doi:10.1016/0092-8674(94)90360-3. 8124721

[41] Saksela K, Cheng G, Baltimore D 1995 Proline-rich (PxxP) motifs in HIV-1 Nef bind to SH3 domains of a subset of Src kinases and are required for the enhanced growth of Nef+ viruses but not for down-regulation of CD4. EMBO J 14:484–491. doi:10.1002/j.1460-2075.1995.tb07024.x. 7859737 PMC398106

[42] Cohen GB, Rangan VS, Chen BK, Smith S, Baltimore D 2000 The human thioesterase II protein binds to a site on HIV-1 Nef critical for CD4 down-regulation. J Biol Chem 275:23097–23105. doi:10.1074/jbc.M000536200. 10807905

[43] Poe JA, Smithgall TE 2009 HIV-1 Nef dimerization is required for Nef-mediated receptor downregulation and viral replication. J Mol Biol 394:329–342. doi:10.1016/j.jmb.2009.09.047. 19781555 PMC2783173

[44] Pizzato M, Popova E, Gottlinger HG 2008 Nef can enhance the infectivity of receptor-pseudotyped human immunodeficiency virus type 1 particles. J Virol 82:10811–10819. doi:10.1128/JVI.01150-08. 18715908 PMC2573167

[45] Garcia JV, Alfano J, Miller AD 1993 The negative effect of human immunodeficiency virus type 1 Nef on cell surface CD4 expression is not species specific and requires the cytoplasmic domain of CD4. J Virol 67:1511–1516. 8437228 10.1128/jvi.67.3.1511-1516.1993 PMC237521

[46] Popov S, Popova E, Inoue M, Wu Y, Gottlinger H 2018 HIV-1 gag recruits PACSIN2 to promote virus spreading. Proc Natl Acad Sci U S A 115:7093–7098. doi:10.1073/pnas.1801849115. 29891700 PMC6142272

[47] Lindwasser OW, Smith WJ, Chaudhuri R, Yang P, Hurley JH, Bonifacino JS 2008 A diacidic motif in human immunodeficiency virus type 1 Nef is a novel determinant of binding to AP-2. J Virol 82:1166–1174. doi:10.1128/JVI.01874-07. 18032517 PMC2224420

[48] Kmiec D, Akbil B, Ananth S, Hotter D, Sparrer KMJ, Sturzel CM, Trautz B, Ayouba A, Peeters M, Yao Z, Stagljar I, Passos V, Zillinger T, Goffinet C, Sauter D, Fackler OT, Kirchhoff F 2018 SIVcol Nef counteracts SERINC5 by promoting its proteasomal degradation but does not efficiently enhance HIV-1 replication in human CD4+ T cells and lymphoid tissue. PLoS Pathog 14:e1007269. doi:10.1371/journal.ppat.1007269. 30125328 PMC6117100

[49] Peters PJ, Bhattacharya J, Hibbitts S, Dittmar MT, Simmons G, Bell J, Simmonds P, Clapham PR 2004 Biological analysis of human immunodeficiency virus type 1 R5 envelopes amplified from brain and lymph node tissues of AIDS patients with neuropathology reveals two distinct tropism phenotypes and identifies envelopes in the brain that confer an enhanced tropism and fusigenicity for macrophages. J Virol 78:6915–6926. doi:10.1128/JVI.78.13.6915-6926.2004. 15194768 PMC421670

[50] Sullivan N, Sun Y, Li J, Hofmann W, Sodroski J 1995 Replicative function and neutralization sensitivity of envelope glycoproteins from primary and T-cell line-passaged human immunodeficiency virus type 1 isolates. J Virol 69:4413–4422. 7769703 10.1128/jvi.69.7.4413-4422.1995 PMC189183

[51] Mirzabekov T, Bannert N, Farzan M, Hofmann W, Kolchinsky P, Wu L, Wyatt R, Sodroski J 1999 Enhanced expression, native purification, and characterization of CCR5, a principal HIV-1 coreceptor. J Biol Chem 274:28745–28750. doi:10.1074/jbc.274.40.28745. 10497246

[52] Akagi T, Shishido T, Murata K, Hanafusa H 2000 v-Crk activates the phosphoinositide 3-kinase/AKT pathway in transformation. Proc Natl Acad Sci U S A 97:7290–7295. doi:10.1073/pnas.140210297. 10852971 PMC16538

[53] Akagi T, Sasai K, Hanafusa H 2003 Refractory nature of normal human diploid fibroblasts with respect to oncogene-mediated transformation. Proc Natl Acad Sci U S A 100:13567–13572. doi:10.1073/pnas.1834876100. 14597713 PMC263854

[54] Dai W, Usami Y, Wu Y, Gottlinger H 2018 A long cytoplasmic loop governs the sensitivity of the anti-viral host protein SERINC5 to HIV-1 Nef. Cell Rep 22:869–875. doi:10.1016/j.celrep.2017.12.082. 29386131 PMC5810964

